# Lens Factor Choice in IOL Power Calculation after Laser Refractive Surgery: The Right Constant for Advanced Lens Measurement Approach (ALMA)

**DOI:** 10.3390/jcm13175186

**Published:** 2024-09-01

**Authors:** Ferdinando Cione, Maddalena De Bernardo, Margherita Di Stasi, Martina De Luca, Rosa Albano, Nicola Rosa

**Affiliations:** 1 Ophthalmological Unit, Department of Medicine, Surgery and Dentistry, Scuola Medica Salernitana, University of Salerno, 84081 Salerno, Italy; fcione@unisa.it (F.C.);; 2 AOU San Giovanni di Dio e Ruggi D’Aragona, 84131 Salerno, Italy

**Keywords:** IOL power calculation, refractive surgery, lens constant, IOL power formulas

## Abstract

**Background/Objectives**: To evaluate the advanced lens measurement approach (ALMA) formula accuracy using different lens constants available on the user group for laser interference biometry (ULIB) and IOL Con platforms. **Methods:** In this retrospective, comparative, case-series study, 150 eyes of 160 patients with previous myopic Photorefractive Keratectomy (PRK) or laser-assisted in situ keratomileusis (LASIK), who underwent uneventful cataract surgery and IOL implantation, were examined. The ALMA formula was evaluated to calculate the refractive prediction error (PE), analysing four different categories of lens constants: both nominal and optimized A-Constant for SRKT, which are available on the ULIB and IOL Con platforms. An additional analysis was carried out in this study, evaluating if a decreased ULIB optimized constant (DUOC) with different fixed factors (−1.2 −1.3 −1.4 −1.5) could improve refractive outcomes. Median absolute error (MedAE) and percentage of eyes within ±0.50 and ±1.00 diopters (D) of prediction error were measured as the main outcomes. **Results:** Comparing the lens factors available on ULIB and IOL Con platforms, the ALMA formula reported a lower MedAE and higher percentages of eyes with a refractive PE within 1.0 D using ULIB nominal constants (all *p* < 0.05). Using DUOC (−1.3), and there was a statistically significant improvement of both MedAE and of the percentages of eyes with PE within ±0.50 D with the ALMA method compared to nominal ULIB constants (all *p* < 0.05). **Conclusions:** The impact of different lens factors in the IOL power calculation after myopic LRS should be carefully evaluated. The ALMA formula, in the absence of optimized constants by zeroing the mean error, should be used by subtracting 1.3 from the optimized ULIB constants available on the IOL Con website. This finding suggests further studies to test which of these constants could work better with the other post-refractive surgery formulas.

## 1. Introduction

Laser refractive surgery (LSR) produces excellent visual outcomes, increasing the number of patients undergoing this procedure exponentially [1] and, consequently, the number of these patients who, in the future, will undergo cataract surgery [2,3]. In the era of “refractive cataract”, these patients expect independence from glasses after surgery, but it is well known that LRS modifies corneal parameters such as curvature and keratometric index, as well as other ocular structures [4]. Therefore, it makes the formulas based on the mean keratometry, created for a population with no corneal alterations, unsuitable for the calculation of the IOL after LRS [5,6]. For this reason, using the IOL power calculation formula specific for LRS eyes is needed.

IOL power calculation plays a crucial role in the era of “refractive cataract” and many IOL power calculation formulas have been published to obtain the most precise postoperative refractive results [7]. Traditionally, these formulas were classified on a generation-based system, but it would be more useful to refer to these methods with a logical approach-based system [7]. On the other hand, in the case of post-LRS eyes, IOL power calculation formulas were generally classified on the mechanism of function, based on the necessity to know pre-refractive surgery data. The first published methods require the knowledge of patients’ clinical history (pre-refractive surgery keratometric values, pre- and post-refractive surgery refraction). Other formulas do not require these historical data, because reconstructing the patient’s clinical history is not always possible and they can be effectively used in a larger number of patients. A third category of IOL power calculation formulas after refractive surgery is represented by methods that use a combination of historical and current corneal data [7]. The first no-history method, called “R-Factor”, was published by Rosa et al. in 2002. To achieve better refractive results, this method involved the use of a correction factor (R Factor) for the corneal radius applied to the SRK/T formula [8]. However, in some cases, this method has led to excessively myopic post-operative refraction, especially in eyes with increased axial length (AL). For this reason, in 2010, Rosa et al. introduced a further correction factor that allows the calculation of any hypo- or hyper-correction that occurred after PRK surgery [9]. On this basis, an updated version of the original R Factor formula, named the advanced lens measurement approach (ALMA), was published in 2020 [9].

ALMA method was generally analysed by using nominal lens constants that are available at the user group for laser interference biometry (ULIB, http://ocusoft.de/ulib/, accessed on 12 June 2024) [9,10], but unfortunately, this online database has not been updated since 31st October 2016 and the current nominal lens constants are obsolete. A more updated lens constants database is available on the IOL Con platform (https://iolcon.org, accessed on 12 June 2024) under the guidance of Achim Langenbucher.

Since it is known that, in normal eyes, the use of optimized constants published on the ULIB and IOL Con platforms improves the IOL power calculation, the impact of these constants in the case of LRS eyes is less known.

This study aims to evaluate the ALMA formula accuracy in calculating the IOL power for myopic LRS eyes using different lens constants available on the ULIB and IOL Con platforms.

## 2. Materials and Methods

### 2.1. Participants and Study Protocol

This is a comparative case series study with a retrospective review of 160 eyes of 160 patients with previous myopic Photorefractive Keratectomy (PRK) or laser-assisted in situ keratomileusis (LASIK) who underwent uneventful cataract surgery and IOL implantation. Regarding the patient database, it was partly derived from our own database and partly derived from freely accessible databases in the literature [11].

The research was in accordance with the Declaration of Helsinki and a written informed consent was obtained from all our patients before cataract surgery. Given the multi-database nature of the study, the final evaluation of the postoperative data was carried out at the Eye Unit, Department of Medicine, Surgery and Dentistry, Scuola Medica Salernitana, University of Salerno. Institutional Review Board (IRB) approval was obtained from the reference institution of the data collection center (Cometico Campania).

Regarding the evaluation of patient data from the literature, as performed in other studies [11], only eyes where biometric and refractive data were available were selected, applying the same inclusion and exclusion criteria to our eyes.

The inclusion criteria were as follows:

- Previous myopic PRK or myopic LASIK;
- Standard phacoemulsification surgery;
- Stable postoperative refraction obtained at least 1 month after cataract surgery.

The exclusion criteria were as follows:

- Unknown preoperative keratometry or AL or refraction data, or AL measurement obtained with ultrasound biometry;
- Unknown postoperative refraction data;
- Unknown model or power of the implanted IOL;
- Previous refractive surgery different from PRK/LASIK (such as radial keratotomy) or non-myopic LRS;
- Corneal diseases that could interfere with keratometric readings reliability (such as severe dry eye, pterygium, corneal ectasia);
- Ocular or systemic pathologies that could interfere with the post-operative refractive result;
- Any intraoperative and postoperative complications;
- Corrected distance visual acuity <20/25;
- Postoperative refraction obtained before 1 month from cataract surgery.

In the preoperative phase, a complete ophthalmological examination was performed, including visual acuity, optical biometry for AL measurement, and intraocular pressure evaluation.

Post-operative refraction was evaluated at least one month after cataract surgery using an objective method (autorefractometer) and refined with a subjective method.

The ALMA formula was evaluated in order to calculate the refractive prediction error (PE) using A-Constant for SRKT, analysing 4 different categories of lens constants:

- The nominal constants published on the ULIB platform;
- The nominal constants published on the IOL Con platform;
- The optimized constants published on the ULIB platform;
- The optimized constants published on the IOL Con platform.

Median absolute errors (MedAE) and percentages of eyes with PE within ±0.50 and ±1.00 diopters (D) were analysed.

Noting that ALMA’s optimized constants through zeroing out the mean error (ME) were always lower than nominal and optimized ULIB constants [9,10], in this study, an additional analysis was carried out to evaluate if a decreased ULIB optimized constant (DUOC) with a fixed factor, and IOL power calculation accuracy could be improved.

### 2.2. Statistical Analysis

Data collection was performed with Microsoft Excel and statistical analysis with IBM SPSS Statistics Version 26 (International Business Machine Corporation, Armonk, NY, USA).

In each group and for each parameter, the following were calculated:

- ME;
- Median absolute error (MedAE), the main outcome of the study;
- Mean absolute error (MAE);
- Number and percentage of eyes within ±0.5 and ±1.0 D of PE, main outcomes of the study;
- Minimum, maximum, and standard error, 95% confidence interval around the ME;
- Interquartile ranges (IQR).

In addition, the following statistical tests were performed:

- Exact Kolmogorov–Smirnov test to determine the normality of the population distribution;
- One-sample T-test for screening whether the ME was significantly different from zero;
- Friedman’s test with Bonferroni correction to compare MedAE;
- Cochran Q test with and without Bonferroni correction to compare the percentage of eyes within ±0.50 D and ±1.00 D of PE;

*p*-values less than 0.05 were considered statistically significant.

The preliminary sample size, which is mandatory in this type of study [12], was calculated with G*Power software (Version 3.1.9.7, Faul, Erdfelder, Lang, & Buchner, 2020). Given a partial η2 of 0.253, a non-sphericity correction of 0.908 corrected with the Huynh–Feldt method, both calculated with SPSS software Version 26, and an effect size of 0.582, it was estimated that a sample size of 55 eyes would be necessary, with a significance level of 1% and a test power of 99%.

## 3. Results

Table 1 shows the IOL models analysed with the different constants and Table 2 shows the biometric parameters of the analysed eyes. For unpublished data, keratometric readings and AL measurements were performed by experienced operators in the routinely used method. In particular, at least three scans were made for keratometry and at least five scans were conducted for AL measurements, utilizing the phakic option available on the biometer. All data, not in absolute values, were normally distributed (*p* > 0.050).

**Figure 1:**
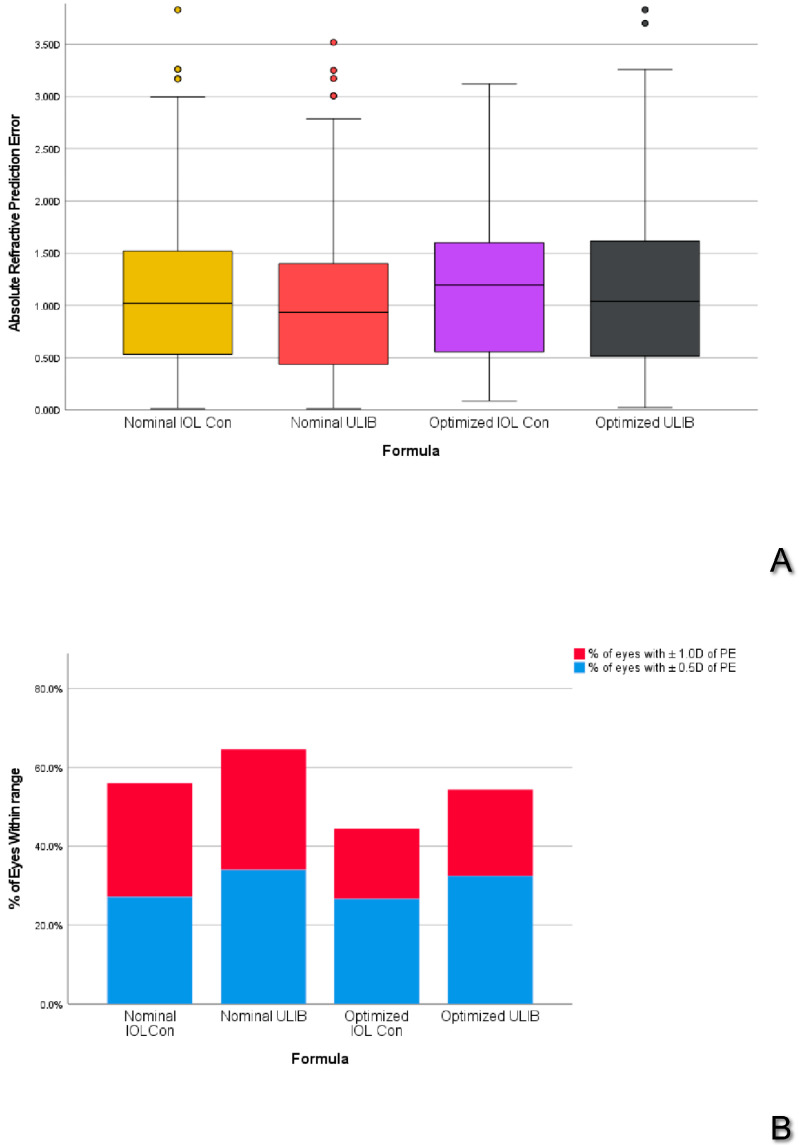
(**A**) Boxplot of comparison between IOL Con and ULIB constants with the ALMA method. (**B**) Percentage of eyes within 0.5 D and 1.0 D with different lens factors with the ALMA method. Thick line: Median; Whiskers: range of non-anomalous values; Dots: mild outlier values.

The analysis of the refractive outcomes obtained by the ALMA formula using different categories of lens constants are shown in Table 3 and in Figure 1A,B.

Because of the little data available for optimized IOL Con constants, the ALMA method with these lens factors was not evaluated through the Friedman and Cochran test.

ALMA formula reported a lower MedAE and higher percentages of eyes with a refractive prediction error (PE) within 1.0 D using ULIB nominal constants (all *p* < 0.05).

Furthermore, considering that the ALMA method produced better results using the lowest lens constants [9,10], refractive outcomes were compared using the ALMA formula with nominal ULIB A-constant and with optimized constant ULIB A-constant, available on the IOL Con platform, by subtracting:

−1;
−1.1;
−1.2;
−1.3;
−1.4;
−1.5.

The optimized ULIB constants available on the IOL Con platform were chosen as a reference for the modified constants because they represent those with the highest number of available data: in fact, only for optimized ULIB constants are there available data of the entire analysed IOL database, and these constants are available on the updated and updating IOL Con website.

Table 4 shows ME’s analysis of the ALMA formula with different modified constants. With DUOC −1.2, −1.3, −1.4, and −1.5, an ME not different from zero was reported, meaning that any systematic error was eliminated [13].

**Figure 2:**
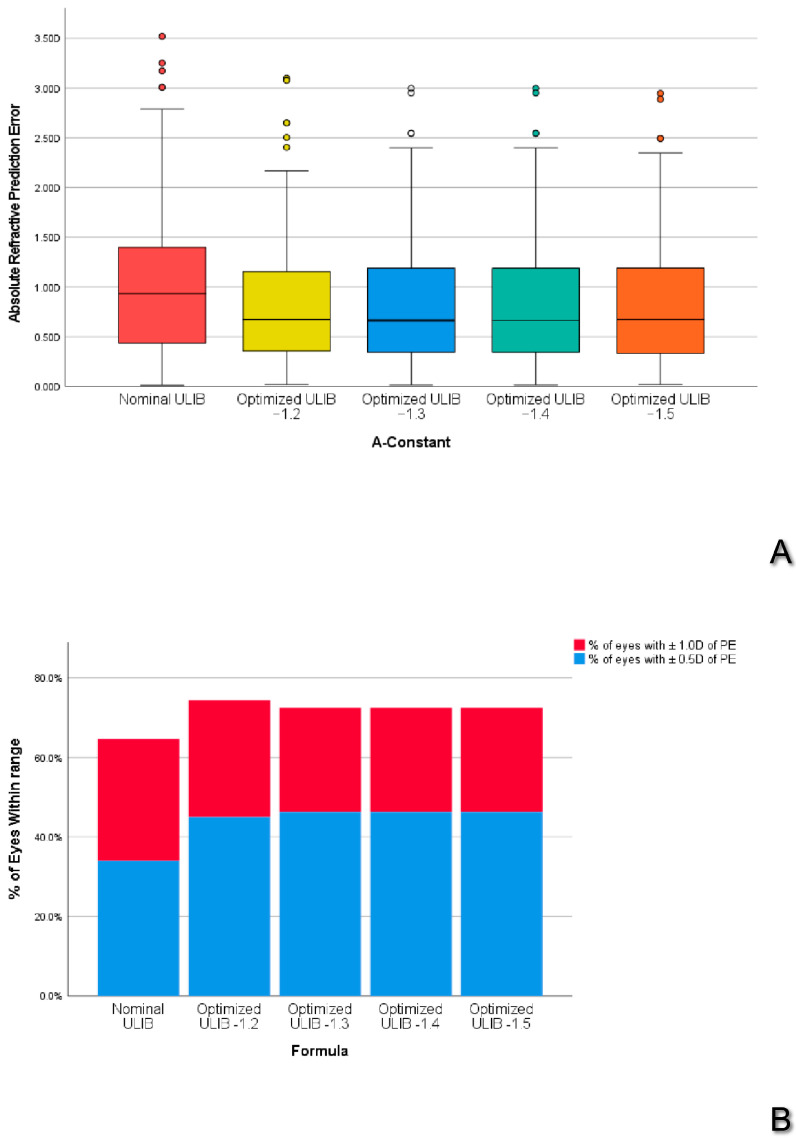
(**A**) Boxplot of comparison between different lens factors with the ALMA method. (**B**) Percentage of eyes within 0.5 D and 1.0 D with different lens factors with the ALMA method. Thick line: Median; Whiskers: range of non-anomalous values; Dots: mild outlier values.

For this reason, PEs obtained with ALMA using DUOC −1.2, −1.3, −1.4, and −1.5 were compared to the ALMA formula using the nominal ULIB constant. Results of such analysis were reported in Table 5 and in Figure 2.

With all DUOC, the ALMA formula showed a statistically significant improvement of MedAE or in the percentages of eyes with PE less than ±0.50 D or less than ±1.00 D compared to ALMA with nominal ULIB constant. Only with a DUOC −1.3 was there a statistically significant improvement in both MedAE and percentages of eyes with PE within ±0.50 D compared to ALMA with nominal ULIB constants.

## 4. Discussion

The presence of a previous LRS in the patient’s clinical history is a very challenging issue for several reasons. In fact, it is well known that PRK and LASIK techniques modify the corneal biomechanics and corneal thickness, making several measurements, such as the intraocular pressure and the corneal power, unreliable [10,14]. But the most important LRS consequence is its impact on IOL power calculation, with the most common IOL power calculation formulas designed for virgin eyes that underestimate the power of the lens resulting in a “hyperopic surprise” [15]. Lots of dedicated IOL power calculation formulas have been proposed, including a “multiformula approach” [10]. Despite this, refractive outcomes after cataract surgery in post-LRS eyes are still worse compared to eyes without a history of refractive surgery. In fact, Ferguson et al. observed in a recent study [16] that in the case of eyes without a history of refractive surgery, over 80% of eyes reached PEs within ±0.5 D; on the other hand, the studies regarding IOL power calculation in post-LRS eyes reported less than 70% within ±0.5 D of the target [2,16,17]. Several reasons have been proposed to explain the lower accuracy of IOL power calculation after myopic LRS [1], but to the best of our knowledge, the lens constant in these eyes was not properly investigated. The role of lens constants optimization to reduce the absolute PE and to obtain a lower postoperative residual refractive error is widely accepted [18,19,20]. Therefore, ophthalmic surgeons should evaluate the refractive results after cataract surgery in order to customize the specific constants with the aim of reducing the refractive error [21]. ULIB database and, more recently, the updated IOL Con platform, offer optimized IOL constants based on thousands of clinical results provided by surgeons and manufacturers from all over the world. Both databases are freely accessible on the following websites: http://ocusoft.de/ulib/ (accessed on 12 June 2024) for nominal ULIB constants and https://iolcon.org (accessed on 12 June 2024) for both optimized ULIB constants and IOL Con constants. Even if the ULIB platform has not uploaded since 31 October 2016, optimized Haigis, Hoffer-Q, Holladay 1, SRK/T and SRKII lens constants are still available. Meanwhile, the IOL Con platform contains not only all ULIB-optimized constants but also new optimized constants for other modern IOL power calculation methods, such as the Castrop formula or the Cooke K6 formula. Both databases collected surgical and refractive data from a vast audience of ophthalmologists from all over the world and the number of analysed eyes for each IOL model is reported on both platforms. This is a great help for the ophthalmic surgeon, but these databases do not distinguish virgin eyes from post-LRS eyes. These different categories of eyes cannot be considered the same thing; for this reason, we consider not optimal the use of optimized lens constants for virgin eyes in IOL power calculation after refractive surgery.

On these bases, we proposed this paper, aiming to detect the best lens constant with the recently published ALMA method, designed for post-PRK or post-LASIK eyes [9]. Therefore, this study was inspired by previous papers in which different constants in the calculation of the IOL were analysed.

In a 2016 study, Chong EW et al. compared the use of different constants in various IOL power calculations by analysing the refractive outcomes using standard formulas (Holladay 1, SRK/T, Hoffer Q, and Haigis) with manufacturers’ ULIB constants, manufacturer modified constants modified with the method of axial length adjustment and with new generation IOL power calculation formulas (Barrett Universal II, Holladay 2 and Olsen) [22]. Better results were obtained with Barrett Universal II (default constants), Haigis (ULIB constants), SRK/T, Holladay 2 (default constants), and Olsen (default constants) in eyes with AL > 26.0 mm and IOL power > 6.0 D. Barrett Universal II formula (default constant), Haigis (axial length adjusted constant) and Holladay 1 (axial length adjusted constant) formulas should be used when AL > 26.0 mm and IOL < 6.0 D.

In the literature, we were not able to find similar studies comparing the accuracy of different constants used in IOL power calculation formulas after LRS. In fact, usually, ULIB or IOL Con constants are indiscriminately chosen in this scenario [23,24,25].

Our study evaluated the refractive outcomes obtained by the ALMA formula using four different lens constants. The manufacturer’s constants available on the ULIB and IOL Con platforms and the optimized constants present on the same databases were evaluated with the purpose of defining the accuracy of each constant in IOL power calculation. ALMA formula reported a lower MedAE using the ULIB manufacturer constants (*p* < 0.05). Since it was demonstrated that the ALMA method works better when the lens constant assumes a lower value [9,10], we verified the improvement of the refractive outcomes by subtracting the values −1, −1.1, −1.2, −1.3, −1.4, and −1.5 from the optimized constant ULIB. Then, we chose the optimized variant with a mean error equal to zero. The optimized ULIB constant, available on the IOL Con platform, has been utilized because it is more updated and most of the data are available on this platform.

Moreover, we found that DUOCs improved refractive outcomes with the ALMA method compared to nominal ULIB constants. In detail, the best results were obtained by using DUOC −1.3. Also, with other DUOCs, there were statistically significant improvements in refractive outcomes, meaning that a residual systematic error, due to the lens constants, was still present. In fact, not only the formula’s accuracy but also other sources of systematic error can result in an unwanted refractive error. Ocular parameter measurement reliability due to optical biometer or eye drop instillation is an example of systematic error [26,27,28], but also an incorrect choice of the lens constant is an important source of systematic error [27,29]. Formula constants interact directly with the effective lens position (ELP) and they are used as tuning parameters to customize the lens power formula to the characteristics of a lens type (to the IOL material and optics/haptics design) [29], but they should be optimized also in relation to ocular parameters, for example, for post-LRS eyes. The reference optimized constants databases do not take into account this difference: our study demonstrated that a residual systemic error persists when using both nominal and optimized A-constants with the ALMA method (which is based on the SRKT formula [10]), as shown in Table 4. Since optimization is the process of finding the specific value of a lens constant that, when used for that particular IOL type, will result in the most accurate IOL power calculations [26], and specific IOL power calculation must be used in case of post-LRS eyes, this paper suggests to also take in consideration previous LRS when performing such optimization in order to propose specifically optimized constants for these specific categories of eyes and IOL formulas.

Both MedAEs and MAEs were reported in this paper, given the debate regarding the primary outcome that should be used in IOL power accuracy studies. Some authors reported that MAE should be more sensitive than MedAE regarding outliers [13]. On the other hand, we preferred MedAE because the distribution of the absolute refractive PEs is not Gaussian and it is described better by the median, as reported also by Hoffer et al. [13]. We reported both values in order to offer the largest amount of data about our study. It should be noted Holladay et al. proposed a different approach in IOL power calculation accuracy studies that is based on the use of root mean square (RMS) and absolute RMS. (RMSAE) parameters [30]. They proposed such a different approach on the assumption that PEs are rarely Gaussian and there are some situations where the ME of PEs is not zero, and it is not suitable to adjust it, for example, in the case of long or short eyes [30]. Regarding the PEs’ distribution, Hoffer et al. have already demonstrated that they are often normally distributed [31], and Table 4 of our study showed that all PEs followed a Gaussian distribution checked by the exact Kolmogorov–Smirnov test. The same table reported MEs equal to zero with the majority of DUOCs, without performing constant optimizations. For these reasons, we believed that our approach based on both MedAE and the percentage of patients with PE within ±0.5 D and ±1.0 D analysis was the more appropriate. MedAE was considered a reliable parameter also in studies that analysed RMSAE [32,33]. A novel protocol analysis that evaluates MEdAE, RMSAE, and percentages of eyes within ±0.5 D and ±1.0 D, together with the IOL Formula Performance Index [13], would be the future in these types of studies.

However, our study shows some limitations: firstly, we did not analyse other IOL power calculation formulas after LRS in order to propose specifically optimized constants for them, but the principal aim of this study was to evaluate the choice of the best constant with the ALMA method. The comparison between the ALMA method with DUOC and other IOL power calculation formulas after myopic LRS is a future perspective, together with the evaluation of the right lens factors in other post-refractive surgery IOL power calculation formulas.

## 5. Conclusions

The impact of different lens constants in IOL power calculation in the case of eyes with a previous refractive surgery should be carefully evaluated because standard optimized constants available on reference online databases can be unsuitable for post-LRS IOL power calculation formulas.

The ALMA method, in the absence of optimized constants by zeroing the mean error on a large patient database, should be used by subtracting 1.3 from the optimized ULIB constant available on the IOL Con platform.

## Figures and Tables

**Table 1:** Lens constant for each analysed IOL model.

IOL Model		A—Constant			
	N°	Nominal ULIB	Nominal IOL Con	Optimized ULIB	Optimized IOL Con
AcrySof IQ Vivity DFT015	1	-	118.80	119.20	119.08
AcrySof TFNT00	2	119.10	119.10	119.10	119.13
Alcon Acrysof MA 60 BM	5	118.90	-	119.80	-
Alcon Restor Sa60D3	2	118.10	-	118.50	-
Alcon SA60AT	3	118.40	118.40	118.80	118.83
Alcon SN60WF	25	118.70	118.70	119.00	118.93
AMO Sensar AAB00	5	118.40	119.00	119.00	118.97
AMO sensar AR 40e	14	118.40	118.70	118.70	-
AMO Tecnis PCB00	21	118.80	119.30	119.30	-
AMO Tecnis Z9000	16	119.00	-	119.20	-
AMO Tecnis ZMA00	2	119.10	119.10	119.50	-
B&L Akreos Adapt	11	118.00	118.00	118.40	-
B&L Akreos AO MI60	2	118.40	118.40	119.10	-
Clareon CNAT00	1	-	118.80	119.10	119.33
Corneal ACR 600 SE	2	119.50	-	120.30	-
Corneal ACR6D	1	118.50	-	119.00	-
Corneal quatrix	1	119.60	-	119.80	-
Curamed SA 60CZ	3	118.80	-	118.50	-
Hexavision HQ 203 HEP	2	118.20	-	118.50	-
Hoya 118,5 AF1FY60AD	3	118.40	118.40	118.60	-
Hoya 250	7	118.40	118.40	118.50	-
Hoya Nc1-Sp (Nanex)	6	-	119.20	119.11	119.11
Hoya va 60 bb	6	118.70	118.70	118.70	-
RayONE	2	-	118.00	118.60	118.70
SN6ATx	7	119.00	119.00	119.20	-
Soleko Fil611	3	118.50	-	119.10	-
Tech Med ISP60H/Z	6	-	118.20	118.70	119.08
Zeiss CT spheris 203	1	118.00	118.00	118.50	119.13

N° = number of IOL for each IOL model. Nominal ULIB constants available at http://ocusoft.de/ulib/ (accessed on 12 June 2024); nominal IOL Con and both optimized ULIB and optimized IOL Con constants available at https://iolcon.org (accessed on 12 June 2024); - = lens constant not available for that IOL model. Only IOL models with the availability of at least two lens constants were evaluated.

**Table 2:** Biometric parameters of analysed eyes.

160 Eyes	Parameter			
	K1	K2	Km	AL
Median	37.82 D	38.36 D	38.07 D	27.53 mm
Mean	37.80 D	38.47 D	38.09 D	27.75 mm
Standard Deviation	2.68 D	2.53 D	2.66 D	2.07 mm
Standard Error	0.21 D	0.20 D	0.21 D	0.16 mm
95% Confidence Interval around the Mean	37.38–38.22 D	38.08–38.87 D	37.67–38.50 D	27.42–28.07 mm
Minimum	30.12 D	32.48 D	29.42 D	23.45 mm
Maximum	44.29 D	45.12 D	44.71 D	34.20 mm
Interquartile Range	3.84 D	3.23 D	3.33 D	2.91 mm
KS	0.982	0.849	0.927	0.553

KS = Normality checks through exact Kolmogorov–Smirnov test; D = diopters; mm = millimiters.

**Table 3:** Refractive outcome obtained with ALMA formula using different lens constants.

A Constant	MedAE (MAE/STD)	P1		SD (95% CI)	IQR (Min–Max)	N°/% <0.5 D	P2		N°/% <1.0 D	P3	
Nominal ULIB N° = 144 eyes	0.94 D (1.02 D/0.06 D)	0.000	A 0.002	0.76 D (0.89–1.14 D)	0.96 D (0.01–3.52 D)	49/34.0%	0.301	-	93/64.6%	0.001	A 0.041
Nominal IOL Con N° = 125 eyes	1.02 D (1.15 D/0.07 D)		B 0.000	0.80 D (1.01–1.29 D)	0.99 D (0.01–3.82 D)	34/27.2%		-	70/56.0%		B 0.000
Optimized ULIB N° = 160 eyes	1.04 D (1.15 D/0.06 D)		C 0.001	0.80 D (1.02–1.27 D)	1.11 D (0.02–3.83 D)	52/32.5%		-	87/54.4%		C 0.102
Optimized IOL Con N° = 45 eyes	1.19 D (1.18 D/0.11 D)	Not Analysed		0.72 D (0.96–1.39 D)	1.08 D (0.09–3.12 D)	12/26.7%	Not Analysed		20/44.4%		

N° = number of eyes.; D: diopters; MedAE: median absolute error; MAE: mean absolute error; STD: standard error; SD: standard deviation; 95% CI: 95% confidence interval around the mean; IQR: interquartile range; Min–Max: minimum and maximum; N°/% <0.5 D/N°/% <1.0 D: numbers and percentages of eyes with a refractive prediction error (PE) within 0.50 D and within 1.00 D. P1: comparison of MedAE through Friedman’s test with Bonferroni correction; P2: Comparison of % <0.5 D through Cochran Q test; P2: comparison of % <1.0 D through Cochran Q test. (P1/P2/P3 calculated on 109 patients). For P1 and P3—A: *p*-value of pairwise comparison nominal ULIB vs. nominal IOL Con; B: *p*-value of pairwise comparison nominal ULIB vs. optimized ULIB; C: *p*-value of pairwise comparison nominal IOL Con vs. optimized IOL.

**Table 4:** Mean error analysis by utilizing different lens factors with the advanced lens measurement approach.

A-Constant		ALMA			
	N°	ME ± SD	Median (STD)	KS	*p*
Nominal ULIB	144	−0.72 ± 1.04 D	−1.02 D (0.14 D)	0.807	<0.001
Nominal IOL Con	125	−0.96 ± 1.02 D	−1.02 D (0.13 D)	0.461	<0.001
Optimized ULIB	160	−0.97 ± 1.00 D	−1.19 D (0.13 D	0.683	<0.001
Optimized IOL Con	45	−1.13 ± 0.79 D	−1.16 D (0.13 D)	0.716	<0.001
Optimized ULIB −1.0	160	−0.30 ± 1.06 D	−0.36 D (0.08 D)	0.905	<0.001
Optimized ULIB −1.1	160	−0.23 ± 1.06 D	−0.31 D (0.08 D)	0.871	0.006
Optimized ULIB −1.2	160	−0.16 ± 1.06 D	−0.24 D (0.08 D)	0.857	0.054
Optimized ULIB −1.3	160	−0.09 ± 1.07 D	−0.18 D (0.08 D)	0.914	0.259
Optimized ULIB −1.4	160	−0.03 ± 1.08 D	−0.10 D (0.09 D)	0.955	0.742
Optimized ULIB −1.5	160	0.04 ± 1.09 D	−0.02 D (0.09 D)	0.955	0.641

N°: number of eyes; D: diopters; ALMA: advance lens measurement approach; ULIB: user group for laser interference biometry; ME: mean error; SD: standard deviation; STD: standard error; KS: normality check through exact Kolmogorov–Smirnov test; *p*: difference from 0 of each ME according to one-sample *t*-test.

**Table 5:** Refractive outcome obtained with ALMA formula using different modified optimized ULIB lens constants, compared to nominal ULIB constants.

A Constant	MedAE (MAE/STD)	P1		SD (95% CI)	IQR (Min-Max)	N°/% <0.5 D	P2		N°/% <1.0 D	P3	
Nominal ULIB N° = 144 eyes	0.94 D (1.02 D/0.06 D)	0.002	A 0.058	0.76 D (0.89–1.14 D)	0.96 D (0.01–3.52 D)	49/34.0%	0.008	A 0.064	93/64.6%	0.034	A 0.031
Optimized ULIB −1.2 N° = 160 eyes	0.67 D (0.84/0.05 D)		B 0.010	0.67 D (0.74–0.95 D)	0.80 D (0.02–3.86 D)	72/45.0%		B 0.006	119/74.4%		B 0.112
Optimized ULIB −1.3 N° = 160 eyes	0.66 D (0.84/0.05 D)		C 0.006	0.67 D (0.74–0.94 D)	0.81 D (0.00–3.94 D)	78/48.8%		C 0.064	118/73.8%		C 0.346
Optimized ULIB −1.4 N° = 160 eyes	0.66 D (0.84/0.05 D)		D 0.007	0.68 D (0.74–0.95 D)	0.84 D (0.02–4.03 D)	74/46.3%		D 0.011	116/72.5%		D 0.346
Optimized ULIB −1.5 N° = 160 eyes	0.68 D (0.85/0.05 D)			0.68 D (0.74–0.95 D)	0.87 D (0.02–4.11 D)	74/46.3%			116/72.5%		

N° = number of eyes; D: diopteres; MedAE: median absolute error; MAE: mean absolute error; STD: standard error; SD: standard deviation; 95% CI: 95% confidence interval around the mean; IQR: interquartile Range; Min-Max: minimum and maximum; N°/% <0.5 D/N°/% <1.0 D: numbers and percentages of eyes with a refractive prediction error (PE) within 0.50 D and within 1.00 D. P1: comparison of MedAE through Friedman’s test with Bonferroni correction; P2: comparison of % <0.5 D through Cochran Q test with Bonferroni correction; P2: comparison of % <1.0 D through Cochran Q test. (P1/P2/P3 calculated on 109 patients). For P1 and P3—A: *p*-value of pairwise comparison nominal ULIB vs. nominal optimized ULIB −1.2; B: 1.3; C: 1.4 D: 1.5. Other pairwise comparisons between optimized ULIB constants were always not significant (all *p* > 0.050).

## Data Availability

The datasets generated and analyzed during the current study are available from the corresponding author upon reasonable request.
